# Health systems research in fragile and conflict affected states: a qualitative study of associated challenges

**DOI:** 10.1186/s12961-017-0204-x

**Published:** 2017-06-07

**Authors:** Tim Martineau, Aniek Woodward, Kate Sheahan, Egbert Sondorp

**Affiliations:** 1 Liverpool School of Tropical Medicine, Pembroke Place, Liverpool, L3 5QA United Kingdom; 2 Public Health and Policy, London School of Hygiene & Tropical Medicine, London, United Kingdom; 3 University of North Carolina, Chapel Hill, NC United States of America; 4 KIT (Royal Tropical Institute), Amsterdam, Netherlands

**Keywords:** Health systems research, Challenges, Fragile states, Conflict-affected states

## Abstract

**Background:**

High quality health systems research (HSR) in fragile and conflict-affected states (FCAS) is essential to guiding the policies and programmes that will improve access to health services and, ultimately, health outcomes. Yet, conducting HSR in FCAS is challenging. An understanding of these challenges is essential to tackling them and to supporting research conducted in these complex environments. Led by the Thematic Working Group on Health Systems in FCAS, the primary aim of this study was to develop a research agenda on HSR in FCAS. The secondary aim was to identify the challenges associated with conducting HSR in these contexts. This paper presents these challenges.

**Methods:**

Guided by a purposely-selected steering group, this qualitative study collected respondents’ perspectives through an online survey (n = 61) and a group discussion at the Third Global Symposium on HSR in September 2014 (n = 11). Respondents with knowledge and/or experience of HSR in FCAS were intentionally recruited.

**Results:**

Of those ever involved in HSR in FCAS (45/61, 75%), almost all (98%) experienced challenges in conducting their research. Challenges fall under three broad thematic areas: (1) lack of appropriate support; (2) complex local research environment, including access constraints, weak local research capacity, collaboration challenges and lack of trust in the research process; and (3) limited research application, including rapidly outdated findings and lack of engagement with the research process and results.

**Conclusions:**

This study shows that those familiar with HSR in FCAS face many challenges in gaining support for and in conducting and applying high-quality research. There is a need for more sustainable support, including commitment to and long-term funding of HSR in FCAS; investment in capacity building within FCAS to meet the challenges related to implementation of research in these complex environments; relationship and trust building among stakeholders involved in HSR, particularly between local and international researchers and between researchers and participants; and innovative and flexible approaches to research design and implementation in these insecure and rapidly changing contexts.

**Electronic supplementary material:**

The online version of this article (doi:10.1186/s12961-017-0204-x) contains supplementary material, which is available to authorized users.

## Background

The World Bank estimates that two billion people worldwide reside in countries distressed by fragility, conflict and violence [1]. Fragile and conflict-affected states (FCAS) lag behind more stable contexts in meeting international health goals [2, 3]. Implementation of well-known health strategies and technologies proves even more difficult in FCAS than in other, equally poor but more stable countries [4]. FCAS typically have limited institutional capacity, often leading to weak health systems. Health systems research (HSR) needs to take this fragility into account in order to contribute to the development of policies and programmes that will improve health outcomes in the short and longer term [4–6].

Although definitions and classifications of fragile, conflict-affected, and post-conflict states vary in the literature and among development agencies, broad consensus defines a ‘fragile state’ as one that fails to maintain rule of law or perform functions essential to meeting the basic needs of its people, including poverty reduction [7–9]. Further, the extent and experiences of fragility vary greatly within FCAS [10]. Many, but not all, fragile states are affected by or emerging from conflict [7]. Conflict is both a precursor to and consequence of ‘fragility’ – a term increasingly used instead of ‘fragile states’ as it is regarded as less political. Conflict-affected countries often experience prolonged periods of relative stability, during which health system strengthening agendas emerge.

Researchers and donors have found HSR in FCAS to be a growing area of interest [11–14]. However, this area of research remains underdeveloped, partly because of its intrinsic challenges. While a recent evidence review of research on health interventions in humanitarian crises found a significant increase in papers on health systems from 2010 onwards, the quality of research was reported as “*questionable*”, possibly due to the fact that it is conducted in “*insecure and unpredictable environments*” [15]. Along the same line, a donor report states that “*measuring and managing results in FCAS raises specific practical challenges and some opportunities beyond those normally encountered in more effective states*” [16]. In addition, there is some evidence regarding research challenges from papers on conflict and emergency situations, including ethical challenges [17–21], access to the field [22–24], security [20, 24], quality of data [15, 21, 25], in-country research capacity [24], and terminology issues [26, 27]. However, these challenges are generally subjective, based on opinions and experiences of these papers’ authors, and they are not specific to HSR.

HSR, a complex and long-term endeavour, entails additional challenges. A better understanding of the challenges involved specifically in HSR in FCAS is essential in order to tackle them and to support and promote higher-quality research in such contexts.

This study consulted a broad range of stakeholders on their perceptions and experiences of the challenges involved in HSR in FCAS. Results presented here were part of a broader study undertaken to identify research needs in FCAS [13], which was led by the Thematic Working Group on Health Systems in Fragile and Conflict Affected States (from here on called TWG).^1^

## Methods

### Study design

This larger study employed a qualitative descriptive approach using five different stages. The first two stages were used to consult on the challenges and are described here. Information on the other stages is published elsewhere in this journal [13]. Similar techniques have been used in prior research priority setting exercises [28–30].

The first stage involved the development of a steering committee (n = 30) and agreement on the methodological approach. Further details on the selection of the steering committee is found elsewhere [13]. The objectives of the second stage of this research were to develop an understanding of health systems research needs and challenges associated with conducting HSR in FCAS via an online survey and a face-to-face group discussion during the Third Global Symposium on HSR^2^ in Cape Town on September 30, 2014. The aim was to consult a wide variety of participants with knowledge of and/or direct experience of HSR in FCAS.

### Online survey

The survey collected basic demographic information. It also elicited personal information such as how the participant heard about the survey, primary work context, experience working in FCAS, and involvement in HSR in FCAS. To facilitate development of the final survey tool, researchers conducted a pilot survey amongst a sub-section of the steering committee of the overall study and then made revisions based on the pilot survey results and participant feedback. Both the pilot and final survey (Additional file 1) were developed and distributed via the Bristol Online Survey tool, which is commonly used by universities in the United Kingdom and ensures participant anonymity.

All contactable people with at least a minimum expertise in health systems in FCAS were eligible to participate in the final survey. The aim was to obtain a sample of approximately 100 people, including a mixture of male and female respondents with a range of experiences (donors, policymakers, academics, international and local implementers) and from a variety of geographical areas (representing different continents and countries, including FCAS).

**Fig. 1:**
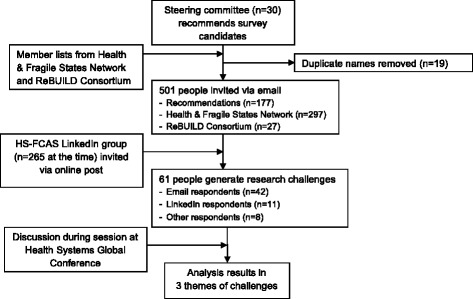
Participant recruitment and data generation process

Researchers recruited respondents through three main channels, namely through (1) the study’s steering committee recommended candidates (n = 177), (2) members of the Health and Fragile States Network^3^ (n = 297) and ReBUILD Consortium^4^ (n = 27) and (3) members of the TWG. The first two groups were asked to participate via an email invitation containing a brief description of the study and a hyperlink to the survey; the third group was invited with a link to the survey through a post on the TWG LinkedIn group^5^ page (which had 264 members, although there was likely a large overlap with those emailed). Figure 1 shows a flowchart of the recruitment and data generation process.

The online survey was open for 2 weeks (October 14–28, 2014) to allow participants to complete it electronically at a private and convenient place. It elicited individual perspectives and did not ask people to respond on behalf of any organisation they work, or have worked, with. The survey, written in English, contained open-ended questions and took about 10–15 minutes to complete. Researchers sent two reminders during the 2 weeks to encourage participation.

### Group discussion

At the symposium, the TWG led the group discussion, which formed one component of the project. During this session, a draft landscaping paper that focused on HSR in FCAS was presented. In addition, two research papers published in a special issue of *Conflict and Health* entitled “Filling the void: Health systems in fragile and conflict affected states”^6^ were presented. Panellists and attendees then discussed health system research needs and the challenges of conducting such research in FCAS.

### Data analysis

One author (AW) transcribed, anonymised and thematically analysed the recorded group discussion. The author also anonymised survey results and analysed quantitative data using Microsoft® Excel® for Mac 2011. Two authors (AW and KS) independently analysed qualitative survey data using thematic analysis with the support of qualitative data analysis software. AW used NVivo for Mac, QSR International Pty Ltd. Version 10, 2014, and KS used ATLAS.ti. version 7.5.11. Each coded the qualitative survey data using inductive descriptive coding [31]. Authors discussed emerging themes via Skype meetings. Both authors identified similar themes, and thus no reconciliation of differences was required.

## Results

The group discussion at the Health Systems Global conference included observations from four panellists and seven session attendees. Discussants, both female and male, represented different professional backgrounds, including academia, funding, non-governmental organisations (NGOs) and policy groups. Discussants did not specify countries of residence.

Sixty-one of 501 contactable and eligible candidates (12.2% response rate) completed the survey. Most (69%, n = 42) heard about the survey via an email invitation: 18% (n = 11) via the TWG LinkedIn group and 13% (n = 8) via another channel such as through a colleague. Overall, 59% of respondents were female and 41% were male, 43% worked in international implementation (e.g. international NGOs), 31% worked in academia (e.g. universities, research institutes), 16% worked in local implementation (e.g. government, local NGO), and 10% worked in funding (e.g. donors).

**Fig. 2:**
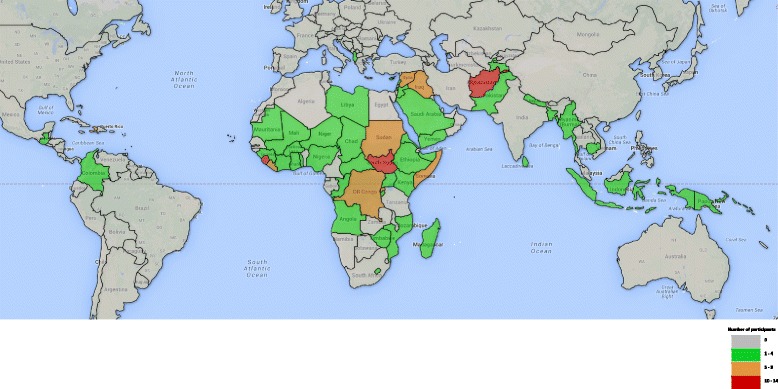
Countries in which survey respondents had professional experience

At the time of the survey, respondents resided in 28 countries, 15 of which were considered by respondents as fragile and/or conflict affected. Most respondents lived in the United Kingdom (12.1%), followed by Afghanistan (8.6%), Sierra Leone (8.6%) and the United States of America (8.6%). Among those with any experience working directly in fragile and/or conflict-affected states (93%), 8.1% had worked in Afghanistan, 7% in South Sudan, 5.8% in Sierra Leone, and 4.1% in Somalia. Respondents might have worked in more than one of these countries. Together, respondents had experience working in 56 different fragile and/or conflict affected countries. Figure 2 7 shows the countries and areas in which respondents had worked. Respondents could list up to five countries. Those who had worked in more than five countries were encouraged to list those in which they had the most experience.

Most survey respondents (92%) felt that the contexts of FCAS are different from the contexts in states not considered fragile or conflict affected. Of those ever involved in HSR in fragile and/or conflict-affected states (45/61, 75%), almost all (98%) experienced challenges in gaining support for, conducting or applying their research.

**Fig. 3:**
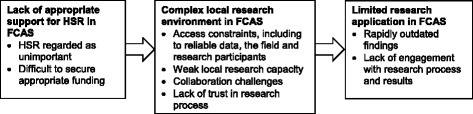
Main challenges encountered during health systems research in FCAS

The identified challenges fell under three broad thematic areas, namely (1) lack of appropriate support, specifically lack of long-term support and commitment, (2) complex local research environment, including access constraints, weak local research capacity, collaboration challenges, and lack of trust in the research process, and (3) limited research application, including rapidly outdated findings and lack of engagement with the research process and results. Figure 3 provides an overview of these challenges as they commonly occur within the research process.

### Lack of appropriate support

Survey and discussion participants articulate a lack of organisational commitment to and support for HSR in FCAS.

First, HSR in FCAS is often regarded as a low priority. One survey respondent working in the academic sector in the United Kingdom noted that it is difficult “*to convince key stakeholders of the importance of doing health systems research*” in FCAS, particularly in post-conflict situations. Numerous respondents echoed this limited interest of key stakeholders such as national governments, international donors and academic institutions.

Second, it is challenging to secure appropriate funding for this area of research. A respondent working in international implementation in the Ukraine stated that “*short funding cycles do not allow for much meaningful research*” and that such short funding cycles make it difficult to discern whether research application has a sustainable impact on the health system. Similarly, discussants at the Cape Town Conference highlighted a lack of funds for comprehensive and longitudinal research in conflict-affected environments. Without long-term support and commitment, it is difficult to conduct meaningful HSR in FCAS. A respondent working in academia and in the funding sector in Iraq confirmed, “*There are no proper resources, continuity, or commitment to maintain the research process in an effective and meaningful manner*”.

Besides limited support and commitment, which are critical to starting and maintaining the research process, the majority of challenges reported relate to the practicalities of conducting HSR in the complex contexts of FCAS. These are discussed below.

### Complex local research environment

Respondents identified access constraints, weak local research capacity, collaboration challenges and lack of trust in the research process as the key factors that present unique, specific and sizeable challenges to conducting HSR in FCAS.

#### Access constraints

Almost all respondents mentioned access constraints as a significant challenge to conducting research in FCAS. These include access to reliable data, the field and research participants.

Many underscored the difficulty of accessing reliable ‘data’, ‘information’ or ‘evidence’ in FCAS. During a crisis, one contributor explained, “*People don’t know the importance* [of data] *at* [the] *national level and international level, and also the donors are more focused on providing services*”. A participant working in academia in the United States added that institutional memory loss, a consequence of “*continual turnover of government and aid agency personnel*” challenges access to information. Further, insecurity limits access to the field. One respondent recounted that travel bans limit access to fragile settings and “*once in-country, safety concerns can limit the ways in which researchers can move and interact*”. Access to research participants is, according to another respondent, challenged by “*poor infrastructure and internet access*”, which makes it “*logistically hard to contact people and arrange interviews*”.

Participants emphasised the need for “*more innovative ways of data collection*” if insecure areas or populations are to be included in research. The constrained access to information, sites and populations might yield a sampling bias. A respondent from Europe working in funding, contributed: “*In FCAS it is difficult to reach the target areas/populations for the research. In more cases, research areas are convenience-selected and based on security and ease-of-access rather than for representativeness of the population/country*.”

#### Weak local research capacity

Many respondents highlighted weak local research capacity in FCAS. Research capacity includes both the necessary staffing (numbers, skills and knowledge) and the infrastructure, for instance, ethical review boards, required for conducting high-quality research.

In terms of necessary staffing, many reported limited in-country capacity. An Afghan respondent working in international implementation experienced a “*shortage of researchers and data collectors for fielding studies*” and “*low capacity of the government and non-government institutions to lead and implement research*” whilst conducting HSR in FCAS. Consequently, the quality of HSR might be compromised. A respondent from Europe working in international implementation remarked: “*Capacity of staff we have found in general to be weaker in conflict affected states. This can affect quality of the research*.” Other respondents cited that research infrastructure is often disrupted or missing in FCAS. A respondent from the United States commented that ethical review boards are “*either non-existent or complicated*”.

#### Collaboration challenges

Respondents identified collaboration as a key challenge to conducting HSR in FCAS. The main reason mentioned was that many different actors are commonly involved in health systems strengthening efforts in FCAS, including research. One respondent recounted a complex consensus-building process that included a variety of actors aiming to build “*one common agenda and support for research*”.

Language barriers, both literal and figurative, can make collaboration difficult. In the figurative sense, national researchers voiced a negative perception of the use of the word ‘fragile’ by international researchers. Further, the use of conceptual language sometimes leads to misunderstandings. A respondent from Sierra Leone who worked on local implementation stated: “*Beyond the issues of conducting any type of health research, which were certainly present, the understanding of the very concepts of some of the systematic issues was not entirely present. So* [these issues were discussed] *with ‘buzz words’, but not with real understanding of the meaning, for many of our team counterparts*.”

#### Lack of trust in the research process

A lack of trust in the research process also stymies collaboration. A survey respondent from Sudan working in academia identified “*a lack of trust between the researchers, participants, and the local community*” as one of the main challenges of conducting HSR in FCAS. The respondent observed the effects of power differentials within the research process, wherein the researcher exercises power by analysing the data provided by participants. Another respondent noted that significant cultural differences resulted in scepticism towards researchers, whom local residents considered “*outsiders*”.

Further, many respondents stated that political and conflict-related sensitivities made research more difficult in FCAS. One respondent noted that mistrust between communities and state and non-state actors makes qualitative research particularly challenging. Another respondent thought that political and conflict-related sensitivities bias research results and therefore affect research quality. A participant from Europe working in international implementation disclosed: “*I interviewed people who had been involved in the health system in South Sudan about health systems strengthening… The main challenges that I encountered were that people still working in South Sudan were often reluctant to speak negatively about the situation as they were worried about repercussions from the government*”. Another respondent mentioned mistrust relating to the “*flow and use of resources*” because, he said, in fragile settings “*this often is partly on a cash basis, and diversions of funds are not uncommon*”.

### Limited research application

Respondents noted that lessons from the HSR conducted in FCAS were not always applied optimally, often because the findings became rapidly outdated and because institutions do not engage substantially with the research process and its results.

#### Rapidly outdated findings

Participants highlighted that contexts within FCAS, including their health systems, change rapidly. A respondent from Europe working in international implementation underscored this, saying: “*The context in a fragile state is much less predictable and constantly changing, meaning that any gains made in the health system and health systems strengthening can be lost overnight, as in the case of South Sudan. This makes research much harder*.” He continued by saying that, because the context is constantly changing, it is more difficult “*to capture the context at any one particular point in time*”. Another respondent noted that, because contexts in FCAS change so rapidly, research findings become out-of-date quickly, which reinforces the reality that research must be “*tailored to each specific situation*”.

#### Lack of engagement with research process and results

Respondents viewed the lack of substantial engagement with the research process and its results as key to poor uptake of HSR. A survey respondent working in Afghanistan explained that national governments were often reluctant “*to engage in health research while other immediate issues, such as security and maintenance of power, are at hand*”. Another factor leading to poor research uptake is lack of “*acceptance of results*” by policymakers. For example, a respondent from Australia working in academia noted that, in the case of health services utilisation analyses, policymakers often regarded health services clients in FCAS as “*unqualified*” providers of information upon which to base policy decisions. Respondents cited a lack of clarity regarding “*whom to disseminate information for translation to policy and practice to*”.

### Group differences

The authors compared respondents by sex and professional background, which resulted in some notable differences. Male survey respondents more often described challenges related to appropriate support for and engagement with the research process and its results than female respondents. Trust issues were raised four times as often by local implementers and almost three times as often by academics than international implementers. Collaboration challenges were highlighted more often by local (almost four times) and international implementers (almost twice) compared to those working in academia. International implementers predominantly raised the issue of limited research application.

## Discussion

This study shows that those familiar with and directly involved in HSR in FCAS face many challenges in gaining support for and in conducting and applying high-quality research. Using an online survey and documenting a discussion group allowed the authors to consolidate and synthesise the experiences of researchers, funders and implementers from a wide range of countries, including many FCAS.

### Contextualising health systems research in FCAS

Results from this study should be contextualised against the background of some broader issues affecting HSR in general and HSR in FCAS specifically. First, HSR is a relatively young field [32] that urgently requires development [33] in order to maximise its contribution to policy and practice. Developing the field is challenging because HSR includes a wide range of complex and interconnected systems and issues (as illustrated by the six WHO building blocks) and is conducted by researchers offering a multitude of diverse disciplinary perspectives [32, 33]. Thus, systematic progression of HSR will take intense effort and coordination across disciplines. Second, the principles for engagement of aid in FCAS increasingly emphasise state building as the central objective [34]. As a result, HSR in FCAS may be critiqued primarily on its contribution to state building rather than on its contribution purely to health outcomes [35]. For this reason, HSR may need to formulate its research more often in relation to fragility, not just being research that happens to be conducted in a fragile state. Finally, the fragile states literature [36] increasingly calls for highly contextualised, flexible approaches for aid in general and for health sector development in particular. This focus demands a highly contextualised and specific evidence base, which may limit research generalisability.

### Recommendations

While this study did not specifically collect data on how to address the challenges commonly confronted when conducting HSR in FCAS, some practical recommendations arise from the data.

#### Need for more sustained support

Health systems strengthening, a lengthy and complex process, should become the primary focus after the transition from humanitarian relief to sustainable development [37]. Evidence suggests that, in addition to improving health outcomes, health systems strengthening may play a significant role in state building by contributing to the reinforcement of government legitimacy [38, 39]. However, because the role of health systems strengthening in influencing health outcomes and state stability in FCAS is insufficiently understood, HSR in FCAS warrants significant and long-term investment.

This study shows that there is currently insufficient institutional support for HSR in FCAS. More funding for HSR in FCAS is required in order to overcome the lack of evidence regarding which health systems strengthening approaches are effective and which are not [40, 41]. Specifically, there is a lack of financial support for and commitment to longitudinal research. Strong longitudinal research is essential to demonstrate the impact of health systems characteristics and strengthening initiatives on health outcomes. More long-term funding would also enable more substantial and nuanced capacity building, and relationship development. Current examples include the ReBUILD Consortium (funded by the United Kingdom’s Department for International Development), which is an international research partnership exploring approaches to health systems development in post-conflict settings [42] and RECAP-SL (funded by the European & Developing Countries Clinical Trials Partnership) that aims to build HSR capacity in Sierra Leone. Seed funding grants may prove useful for testing the feasibility of high-risk research in FCAS.

Institutional support extends beyond financial support in enabling researchers to conduct research even in extremely challenging environments. While HSR in FCAS often entails security considerations beyond those normally confronted by research institutions, overly constrictive travel bans or restrictions imposed by institutions, such as universities, limit the development of contextual knowledge, relationships and the research process. We must understand the nature and effects of fragility in order to strengthen health systems within it. Meaningful support for the research process even in light of the pervasive challenges is critical to high-quality HSR in FCAS.

#### Need for research capacity building

This study shows that investment in research capacity building within FCAS is essential to meet the challenges related to implementation of high-quality research in these complex environments. The study further reinforces the notion that capacity building should not be limited to individual research personnel but should extend to essential research organisations and functions such as universities and ethical review boards [43]. Professional development and retention of skilled people is particularly challenging in fragile situations, where factors that push people out of their environment, such as active conflict, extreme lack of resources and poor leadership, are extremely strong. However, these factors lose intensity in post-conflict situations, thus enabling policies aimed at building and retaining skilled workers to become more relevant [44]. Capacity building undertaken during times of relative stability can enable research to continue during times of instability.

Capacity building is necessary not only because research-related knowledge and skills may be limited as a consequence of disrupted or destroyed education systems, but also because access to FCAS is commonly constrained. In-country authorities or researchers’ universities or employers might impose travel bans or restrictions. The security context may be too poor to allow travel. Such constraints may limit research to more stable areas, leading to sampling bias. Capacity building addresses this challenge by enabling researchers based in less stable areas to conduct their research. For example, during the Ebola epidemic, the United Kingdom-based ReBUILD Consortium researchers were unable to travel to Sierra Leone. However, due to capacity-building efforts aimed to empower researchers to work more autonomously since the programme’s inception, the United Kingdom-based team was able to provide their support remotely while the Sierra Leone-based team successfully carried out the research [42]. Encouraging any researchers to continue work during conflict requires assessing risks and benefits. Reflection upon the necessity and feasibility of the research is recommended [45]. That said, enabling researchers who live in FCAS to conduct research even in the midst of fragility offers great potential for timely research that truly captures the context in fragile environments.

#### Need for relationship and trust building

In addition to a need for capacity building within FCAS, this study also shows a need for relationship building among stakeholders, including local communities, government authorities and international researchers. Often overlooked, relationship building is essential to address challenges that arise from a lack of trust in the research process. A growing body of literature suggests that strengthening trust is not just important in research relationships, but is also critical to other societal relationships that are part of health system strengthening after conflict [46]. Our study shows that HSR in FCAS would particularly benefit from increased investment in relationships among local and international research team members as well as between researchers and research participants.

Interestingly, local implementers in this study more commonly raised trust issues than international implementers, donors or academics. Local implementers may more easily recognise this as an issue because management structures within international organisations often empower international staff more than local staff. Local staff may thus feel wary of international stakeholders. Tensions between local and international stakeholders are common in fragile states. In the context of service delivery, tensions between local government and international organisations have been well documented [37, 47]. One could assume that similar tensions exist in the context of research. Research capacity building may redress power imbalances between local and international stakeholders, and improved communication between both parties may increase mutual understanding and trust.

The lack of trust in the relationship between the international researcher and local research participants or communities might exist for similar reasons. However, research brings an additional element that local participants might perceive as a disadvantage, namely that researchers generally hold the power to interpret data collected during the research process. These interpretations may assert considerable influence over policies and programmes that directly affect participants’ lives. Research in FCAS requires adapting communication techniques, especially if the subject matter relates to politics or conflict and how these may impact people’s lives. Social systems and hierarchies are fluid during conflict and previous studies may not adequately inform research in a new setting or time [48]. This highlights the need for novel approaches to research and the communication of research results [49]. In order to build trust and maximise opportunities to apply research results, it is important to communicate results in a timely fashion not only to policymakers but also to communities. Participatory action research – an approach that involves research participants in the process and therefore equalises the power balance in the research relationship [50] – might be an appropriate solution to address certain research questions.

#### Need for innovative and flexible approaches

This study reinforces the need for innovative and customised approaches to research design and implementation. Innovation is commonly defined as the use of new ideas or methods. What is novel to one context, however, might not be novel to another. Therefore, the authors suggest that an effective question with which to start is ‘What ideas and methods effectively used to facilitate HSR in non-fragile contexts might be transferable to fragile ones?’ Zwi et al. [18] suggest that more active participation of refugees and communities in the research process in conflict situations could be promoted.

Researchers and other stakeholders should consider the value of a range of research methods in FCAS. In many cases, descriptive research would be a good starting point. Longitudinal analysis is essential to HSR because it enables analysis of trends and change in personal and organisational behaviour [48]. However, alternative research methods may also yield valuable results. Although longitudinal quantitative methods generally allow statistical testing and prediction modelling, large-scale longitudinal data collection may not be feasible in fluid and insecure environments. Cross-sectional data collection may be more feasible while qualitative methods may be more adaptable and deliver rich, reliable information. Mixed methods research that combines cross-sectional quantitative and qualitative methods may provide well-rounded insight.

The increased availability of information and communication technology, known as eHealth, has shown potential to improve healthcare in developing countries [51], and could be exploited for research purposes. By using an online survey, this study is an example of the opportunity offered by technology. However, the use of technology in research may introduce bias because of the differing levels of access to it [52]. Marked differences in access to technology exist in FCAS, where most people lack access to even the most basic utilities. Future innovations in research should be designed to include communities and vulnerable people within them. Further, they should be designed in such a way that prevailing health inequities are not exacerbated.

Innovative methods of communicating research results should be pursued. Traditional mechanisms, such as academic journals, may not be accessible to decision-makers in FCAS. Research results may have more impact on policy and practice, from the national level to the household level, if communicated quickly, often and through numerous channels. For example, the Afghan Research and Evaluation Unit, an independent research organisation based in Afghanistan, mobilises these principles in communicating research results [53].

The study findings highlight that this need for innovation requires flexibility on the part of research stakeholders, including funders and researchers themselves. This is consistent with previous studies on research in conflict situations [22, 54]. As Barakat and Ellis point out in a discussion paper on research in war circumstances, “*Where information is hard to come by, one must do everything possible to encourage chance learning*” ([22], p. 153). Giving the example of researchers benefitting from ad-hoc conversations, the authors indicate that such ‘chance learning’ opportunities are “*met with little enthusiasm within the realms of rigid research protocol*” ([22], p. 153). Thus, making room for flexibility in terms of funding, design and implementation of HSR allows researchers to respond to changing environments and needs.

### Limitations

This study has several limitations that should be noted when interpreting these findings. First, participation in both the survey and group discussion was based on self-selection. This meant that, although participation required a minimum of experience in HSR in FCAS, this was not corroborated.

Second, there was a lower than expected survey response rate. Survey non-response is a common source of error in survey-based research [55] and this is reflected here. We have documented potential biases that might have arisen from this. A possible reason for non-response is that, at the time of the survey, the Ebola crisis in West Africa was at its peak. It is likely that many potential respondents were actively engaged in Ebola response initiatives, which could have made our target group less responsive to our survey request. Despite a smaller sample size than anticipated (61 instead of 100), data reached saturation as participants across the sample reported similar challenges.

Third, for feasibility reasons, our survey was only available in English and not in any other languages, which could have prevented some candidates from participating.

Fourth, participation in the online survey was reliant on computer and Internet access and familiarity and therefore could have excluded some potential participants (e.g. those from areas in FCAS with limited Internet connection and/or those with limited computer skills).

Fifth, while men and women were relatively equally represented in the online survey, less balance was achieved in relation to work and geographical background – most survey respondents resided in the United Kingdom and were international implementers or academics and thus their views were over-represented in the results.

Finally, the design of this study was guided by its primary aim of developing a consultative research agenda on HSR in FCAS. Identifying challenges in carrying out research in the context of FCAS was a secondary aim. Consequently, participants were primarily targeted based on their familiarity with HSR and not necessarily on their experience with the obstacles of implementing such research. Additionally, this may have limited the depth of the information provided on challenges involved in conducting HSR in FCAS. Future qualitative research on this topic should consider using data collection methods, such as in-depth interviews, that encourage respondents to provide more detailed information.

The potential biases associated with our sampling methods (purposive snowball sampling) and response rate may limit the generalisability of our findings. We did manage to get a good coverage of country experiences (together, participants had experience working in 56 different fragile and/or conflict-affected countries). While these participants self-defined these as fragile and/or conflict affected, the majority are also listed in popular indices [56, 57].

## Conclusion

Strong and resilient health systems are critical to population health, yet little is known about how to effectively strengthen health systems in FCAS. The quality of research in FCAS has been previously recognised as poor [15, 21, 25], partly due to its associated challenges [24]. HSR is a complex endeavour even in stable environments with a strong research capacity. Undertaking such research in FCAS is therefore challenging, as confirmed by this study.

This paper provides a better understanding, through wide consultation, of what these challenges are. While recommendations are given on how to overcome some of the challenges identified, additional research on the facilitators of HSR is needed. Further, sharing of best practices will be beneficial. Simply knowing that these challenges are common, perhaps unavoidable, may help stakeholders to remain committed to the research process. More long-term commitment to and investment in health systems in FCAS is crucial to inform the policies and programmes that will increase access to health services and ultimately improve the health outcomes for millions of vulnerable people worldwide.

## Additional file

Additional file 1: Online survey. This file shows the survey questions related to challenges from the online survey. (PDF 110 kb)

## Abbreviations

FCAS: fragile and conflict-affected states
HSR: health systems research
NGO: non-governmental organisation
TWG: Thematic Working Group

## Footnotes

1 The TWG-HS-FCAS is a cooperation between the Health & Fragile States Network and the ReBUILD Consortium. As a working group of Health Systems Global, we aim to strengthen HSR in FCAS. http://healthsystemsglobal.org/twg-group/8/Health-Systems-in-Fragile-and-Conflict-Affected-States/.

2 Third Global Symposium on HSR, organised by Health Systems Global, took place in Cape Town (2014), building on two previous symposia held in Montreux (2010) and Beijing (2012). http://healthsystemsresearch.org/hsr2014/home?qt-programme_at_a_glance=1.

3 The Health and Fragile States Network, established in 2007 and consisting of practitioners, researchers and funders, aims to contribute to the evidence base on health and fragile states; to influence policy and advocate collectively on a range of issues which impact on health in fragile states; and to support dialogue, debate and networking amongst those working in health and fragile states, and with those who work in other related sectors.

4 The ReBUILD Consortium conducts research for stronger health systems post conflict. www.rebuildconsortium.com.

5 The TWG LinkedIn group brings together key actors on health in FCAS and promotes research, policy and advocacy actions to develop and implement responsive and context-specific health systems. https://www.linkedin.com/groups/6611870.

6 In cooperation with the TWG, BioMed Central’s journal *Conflict and Health* published a series of articles entitled ‘Filling the void: Health systems in fragile and conflict affected states’. http://www.conflictandhealth.com/series/Filling_the_void.

7 This interactive map was developed via ‘Google - Map Data’ and can be accessed here: https://fusiontables.google.com/DataSource?docid=1nFPsjV6slJjg0v0uPaktlypYzPVc-5sYgDWHLpPD#map:id=3.
