# Supplementary material for: Health systems research in fragile and conflict affected states: a qualitative study of associated challenges
Source: Health Res Policy Syst. 2017 Jun 7;15:44. doi: 10.1186/s12961-017-0204-x (PMC5461673; doi:10.1186/s12961-017-0204-x)
Supplement: Additional file 1: — Online survey. This file shows the survey questions related to challenges from the online survey. (PDF 110 kb) [file 12961_2017_204_MOESM1_ESM.pdf]

Health system research in fragile and conflict affected states

Health Systems in Fragile and Conflict Affected States
a subgroup of Health Systems Global

Health system research in fragile and conflict affected states

Health system research in fragile and conflict affected states

There is no consensus in the research literature on what is meant by *fragile and conflict affected states* (and what countries are included) or *health systems research*. However for the purpose of this survey, this is what is commonly meant by these terms:
- *Fragile and conflict affected states* usually include countries that have been or currently are in conflict and/or have governments who lack the capacity or willingness to perform their core functions such as healthcare provision.
- *Health systems research* usually addresses different aspects of the health system (leadership/governance, health financing, health workforce, drugs/technologies, service delivery) in order to improve coverage, quality, access, safety and equity of health systems, and ultimately population health.

1. Based on your experience and familiarity with research literature, do you think that health system research in fragile and conflict-affected states is **different** from health systems research in other settings?

- ○ Yes
- ○ No
- ○ Don't know

Why yes/no? *(Optional)*

2. Have you **ever been involved** in health systems research in fragile and/or conflict affected states?

- ○ Yes
- ○ No
- ○ Don't know

If 'yes' did you experience **any challenges** in conducting your research?

- ○ Yes
- ○ No
- ○ Don't know

i. If 'yes' what **kind of challenges** did you face?

Continue >

Survey testing only
Check Answers & Continue >
